# Organochlorine Pesticide Residues in Raw Milk Samples Collected From Goat's Dairy Farms of Kastamonu Province, Türkiye

**DOI:** 10.1002/vms3.70955

**Published:** 2026-04-25

**Authors:** Sedat Gökmen, Nurcan Demir

**Affiliations:** ^1^ Department of Pharmacology and Toxicology Faculty of Veterinary Medicine Kastamonu University Kastamonu Türkiye; ^2^ Faculty of Veterinary Medicine Kastamonu University Kastamonu Türkiye

**Keywords:** gas chromatography‐mass spectrometry, goat milk, Kastamonu, organochlorine pesticides

## Abstract

**Background:**

Organochlorine pesticides (OCPs) are persistent environmental contaminants that can accumulate in animal‐derived foods and pose potential risks to human health. This study aimed to investigate the presence, concentration, and potential health risks of selected OCP residues in raw goat milk collected from dairy farms in Kastamonu, Türkiye.

**Methods:**

Raw goat milk samples were collected from 20 goat farms located in urban and rural areas of Kastamonu province. A total of 23 OCP compounds were screened using gas chromatography–mass spectrometry (GC–MS). Detected residues were compared with established maximum residue limits (MRLs), and potential non‐carcinogenic health risks were assessed using hazard quotient (HQ) and hazard index (HI) values.

**Results:**

Among the analyzed compounds, β‐endosulfan, methoxychlor, and p,p'‐DDE were detected in the samples. Methoxychlor was the most frequently detected compound, present in 70% of the samples, with concentrations exceeding the MRL in 13 of the 14 positive samples. β‐Endosulfan and p,p'‐DDE were detected in four samples each but remained below their respective MRLs. A statistically significant difference between urban and rural farms was observed only for β‐endosulfan (*p* = 0.015). Health risk assessment indicated that HQ values for β‐endosulfan (0.0031), methoxychlor (0.0159), and p,p'‐DDE (5.29 × 10^−^^5^) were all below the acceptable risk threshold (HQ < 1), with a total HI of approximately 0.019.

**Conclusion:**

Although OCP residues were detected, the estimated exposure levels suggest no significant non‐carcinogenic health risk for consumers. However, the detection of banned pesticides highlights their environmental persistence and emphasizes the need for continuous monitoring and environmental surveillance.

## Graphical abstract

This study analyzed 20 goat milk samples from Kastamonu, Türkiye, detecting methoxychlor, β‐endosulfan, and p,p'‐DDE residues. While methoxychlor exceeded maximum residue limits (MRL) in 13 of 14 positive samples, other residues remained below established thresholds. Despite these findings, health risk assessments—including hazard index (HI) calculations—indicated that the current residue levels do not pose a significant health risk to consumers.

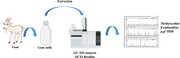

## 1 Introduction

Milk and dairy products are commonly used in almost all societies. It plays a pivotal role in the human diet, regardless of consumers’ age. Milk is acquired from different animal species from different geographical regions (Park and Haenlein 2013). In Türkiye, cow milk is the most produced and consumed milk; however, in recent years, there has been increasing interest in the production and consumption of goat milk and goat milk products.(Güney 2019; TÜİK 2025; Tütenk et al. 2022). In Türkiye, raw milk production in 2024 totalled 22,487,757 tons, comprising approximately 93.6% cow milk (21,040,442 tons), 4.0% sheep milk (906,645 tonnes), 2.1% goat milk (482,247 tons), and 0.3% buffalo milk (58,122 tons) (TÜİK 2025). Goat milk is usually perceived to be more beneficial to health than cow's milk. Proteins in goat milk are more easily assimilated than those in cow milk (Witczak et al. 2016). Goat milk is the closest to human breast milk (Jia et al. 2020). Similar to other animal‐derived products, goat milk has a high risk of contamination by xenobiotics, such as persistent organic pollutants (POPs), metals, mycotoxins, antibiotics, and pesticide residues, despite its many beneficial properties (Baydan et al. 2017; Güvenç and Aksoy 2010).

POPs are industrial compounds and major global environmental contaminants. Organochlorine pesticides (OCPs), polychlorinated biphenyls (PCBs), and polycyclic aromatic hydrocarbons (PAHs) are the most abundant POPs in different environments (air, water, soil, etc.), food chains (milk, eggs, etc.), and biological samples (blood, urine, milk, etc.) owing to their persistence in nature (Guo et al. 2019; Singh et al. 2021). Among these, the primary focus has been on OCPs because of their persistence, stability, lipophilicity, and tendency for bioaccumulation (Liu et al. 2025; Lv et al. 2024). OCPs are divided into three general categories: hexachlorocyclohexanes (which include lindane only), dichlorodiphenylethanes (which include DDT, DDD, and dicofol), and chlorinated cyclodienes (which include Aldrin, Dieldrin, and heptachlor). These chemical compounds accumulate as residues in all food chains, ranging from the soil to animals (Baratzhanova et al. 2024). Farm animals, such as cows and goats, are exposed to OCPs by ingesting contaminated feed (forage and fodder offered to animals), drinking water, or soil partly ingested during grazing (van der Fels‐Klerx et al. 2024). In the Kastamonu region, agriculture and animal husbandry are among the dominant economic activities, and pesticide use in crop production remains relatively common. The region is characterized by mixed farming systems, where livestock production is closely integrated with crop cultivation. Consequently, goats may be exposed to pesticide residues through contaminated pasture, feed crops, soil, and water sources (Icli and Kahyaoğlu 2020). These compounds (lipophilic pesticides) are easily absorbed from the digestive system into the systemic circulation and accumulate mainly in the liver, adipose tissue, brain, kidneys, and milk (Tsiplakou et al. 2010). Moreover, bioconcentration and bioaccumulation of pesticide residues occur along the food chain. The presence of pesticide residues in ruminant milk is a public health concern owing to the widespread consumption of milk and dairy products worldwide. Therefore, in line with the guidelines established by the World Health Organization (WHO), many countries have implemented regulatory frameworks that set maximum residue limits (MRLs) for pesticide residues in milk and dairy products to safeguard public health (Tsiplakou et al. 2010; WHO 2023). OCPs are characterized by their long‐standing persistence and toxicity. Exposure to even low concentrations of these chemicals can cause many adverse health effects in humans, including cancer, fertility disorders, hormonal disorders, and reduced intelligence (Gupta 2004). Given the importance of milk and dairy products in the human diet, consumer exposure to OCP residues in milk may increase health risks.

In Türkiye, many studies have examined pesticide residues, such as OCPs, in raw bovine and sheep milk; however, studies specifically focusing on goat milk are very limited. To ascertain the potential risk to the health of consumers, the present study aimed to determine the OCP residues and assess the associated health risks from raw goat's milk in the Kastamonu Region of Türkiye.

This study was conducted to investigate the presence of organochlorine pesticide (OCP) residues in raw goat milk samples collected from Kastamonu, Türkiye, and to evaluate the related health risks to consumers through risk analysis calculations.

## 2 Materials and Methods

### 2.1 Animals and Sample Collection

Twenty farms, encompassing more than 50 dairy goat herds, were selected from the Kastamonu region of Türkiye for this study. The Turkish Saanen, Angora, and Hair goat breeds (Hair goat's crossbred) were aged approximately 1–4 years on a farm where milking was done automatically or manually. From 20 goat farms, milk samples (500 mL each) were collected between May and August 2024, about 2–3 months after parturition. Milk samples were taken from the bulk milking tank after all goats on the farm were milked. The distance between farms in the selected research locations was at least 40 km. The farms from which milk samples were taken represent the common conventional animal production farms in Kastamonu, Türkiye. The nutrition of the goat herds was based on limited pasture grazing, supplemented by additional feeding from May to August 2024. Goats grazed wooded pastures dominated by shrubs and trees. Supplementary feed consisted of alfalfa hay, wheat straw, and concentrates. The samples were collected in sterile polyethylene containers and transported to the laboratory in insulated refrigerated coolers maintained at approximately 4°C. Upon arrival, the milk samples were stored at −20°C until analysis. All samples were analyzed within one week of collection to minimize any potential degradation of pesticide residues.

### 2.2 Chemicals

The pesticide standard OCP Mix 71 (aldrin, endrin, cis‐chlordane, trans‐chlordane, alpha‐HCH, beta‐HCH, gamma‐HCH (Lindane), o,p'‐DDD, p,p'‐DDD, o,p'‐DDE, p,p'‐DDE, o,p'‐DDT, p,p'‐DDT, methoxychlor, heptachlor, cis‐heptachlor epoxide, trans‐heptachlor epoxide, hexachlorobenzene, dieldrin, alpha‐endosulfan, beta‐endosulfan, technazene, quintozen) was acquired from the German company Dr Ehrenstorfer. For sample extraction and clean‐up, the Q‐sep QuEChERS Q110 and Qsep QuEChERS DSPE Clean‐up kits were acquired from RESTEK Türkiye, and acetonitrile was used as the extraction solvent. All chemicals used in the analysis were of chromatographic grade and purchased from Isolab (Germany).

### 2.3 Sample Preparation

#### 2.3.1 Extraction

The pesticides were extracted using the modified QuEChERS method based on the AOAC official method (Lehotay et al. 2005) with some modifications (Ramezani et al. 2022). Before weighing the samples, the bottles were shaken to ensure good homogenization, and 15 mL of the milk sample was placed in 50 mL Falcon tubes. Subsequently, 15 mL of acetonitrile (1%) was added, and the tube was vigorously shaken for 3 min. The contents of the Q‐sep Q110 QuEChERS kit, containing 4 g of magnesium sulphate (MgSO_4_), 1 g of sodium chloride (NaCl), 1 g of trisodium citrate dehydrate (Na_3_C_6_H_5_O_7_ · 2H_2_O), and 0.5 g of disodium hydrogen citrate sesquihydrate (C_6_H_8_Na_2_O_8_), were added to the tubes. The tubes were vortexed rapidly for 2 min and then centrifuged at 3500 rcf for 5 min in a refrigerated centrifuge.

#### 2.3.2 Clean Up

The supernatants formed in the centrifuged tubes at the extraction step were taken in 15 mL Falcon tubes containing the Q‐sep QuEChERS dSPE mixture (octadecyl C18) and shaken for about 2 min, and then the tubes were centrifuged at 5000 rcf for 2 min in a cooled centrifuge. The supernatant formed was taken with a syringe, passed through a 0.25 µm microfilter, and taken into vials. Finally, the vials were placed in the autosampler of the gas chromatography–mass spectrometry (GC–MS) device, and 2 µL of the sample was injected into the GC–MS system.

#### 2.3.3 Chromatographic Analysis

OCP residue analyses of milk samples were performed at Kastamonu University, Central Research Laboratory Application, and Research Center for Service Procurement. Samples were analyzed using a gas chromatography (GC) system coupled with a mass detector (MS) (Shimadzu QP 2010 ultra brand, Kyoto, Japan). A capillary column (RTX‐5MS, 0.25 µm × 0.25 mm × 30 m, Bellefonte, USA) was employed for analyte separation. Helium served as the carrier gas, and a constant flow rate of 1 mL/min was maintained. The injection block temperature was set to 280°C, the injection mode was splitless, and the injection volume was 2 µL. The column oven program followed a sequence of temperature settings: an initial temperature of 60°C was maintained for 5 min, then increased from 60°C to 280°C with an increase of 5°C/min, and waited for 6 min at 280°C, with a total analysis time of 55 min.

#### 2.3.4 Quality Assurance

Calibration solutions were prepared by diluting the OCP Mix Standard (from Pesticide‐Mix 71) with n‐hexane at six concentration levels (5–200 ng mL^−^^1^), yielding calibration curves with *R* ^2^ values above 0.99. The method performance, limit of quantification (LOQ), precision, and accuracy were validated via recovery studies. According to SANTE/11312/2021 Guidelines (European Commission 2021), LOQs ranged from 0.59 to 4.95 ng mL^−^^1^, with acceptable recoveries (70%–120%) and RSD ≤ 20% for all target compounds.

### 2.4 Calculation of Risk Assessment

Health risk assessments were performed using the hazard quotient (HQ) and hazard index (HI) methods. When HQ < 1, the risk is considered acceptable and does not constitute a long‐ or short‐term health threat. The higher the HQ value, the greater the exposure risk (Wu et al. 2021).

To assess health risks, the lifetime average daily dose (LADD), estimated daily intake (EDI), and hazard quotient (HQ) for pesticides were calculated. To determine the estimated daily intake (EDI) or lifetime average daily dose (LADD), Equation 1; (Witczak and Pohoryło 2015) and Equation 2; (Witczak et al. 2016) were used. According to the latest statistics, the average daily milk consumption of Türkiye is 0.186 kg per person. An average weight of 70 kg was considered for the adults (Akkaya et al. 2025).

(1) EDIorLADD(mg/kgday)=C(mg/g)×CR(g/day)/BW(kg),

(2) HQ=LADD/RfD,

where *C* is the average concentration in milk during the exposure period, CR is the milk consumption rate (0.186 g·person^−1^·d^−1^) for adults, BW is the body weight, average body weight over the exposure period (70 kg male adult), and RfD is the oral reference dose for intake of a particular compound (mg·kg^−1^d^−1^) [β‐endosulfan (0.006), methoxychlor (0.005), and p,p′‐DDD (0.5)] (Witczak et al. 2016).

### 2.5 Cumulative Risk Assessment

The hazard index (HI) was calculated to assess the cumulative effects of the chemicals according to Equation (3):

(3) HI=∑HQ,

where HI values <1 are considered acceptable and do not constitute a health threat in the short term, whereas values >1 pose an unacceptable risk (Wu et al. 2021).

### 2.6 Data Evaluation

All data analyses were performed using the SPSS software (version 23.0; SPSS Inc., NY, USA). The results of the residue concentrations of organochlorine compounds in milk samples are presented as means and standard errors (SE). The significance of contamination in goat milk samples from urban and rural areas was assessed using a *t*‐test. For the calculation of *p*‐values for goat milk in urban and rural areas, it was assumed that undetectable data were half of the detection method limit (Aydin et al. 2019). Significant differences between the urban and rural goat milk samples were identified at *p* < 0.05. In addition, the residue level was evaluated according to the “Turkish Food Codex Regulation on Maximum Residue Limits of Pesticides.”

## 3 Results

### 3.1 Analytical Parameters

Solutions of the compounds at different concentrations were injected into the device, calibration curves were drawn, and the coefficient of determination (*r* ^2^) values were calculated. The limits of detection (LOD) and limits of quantification (LOQ) for the analyzed OCPs were also determined. The blank (pesticide‐free) milk used for the recovery studies was subjected to extraction and cleaning steps and analyzed using a GC–MS system. The *r* ^2^, LOD, LOQ, and percent recovery values of the compounds are presented in Table 1.

**TABLE 1:** *r* ^2^, LOD, LOQ and recovery value of OCPs.

OCPs compounds	*r* ^2^	LOD	LOQ	Recovery %
Tecnazene	0.9995	0.509	1.695	104.652
alpha.‐Lindane	0.9996	2.049	6.829	101.914
Hexachlorobenzene	0.9998	2.423	8.078	102.265
Beta.‐Hexachlorocyclohexane	0.9993	4.107	13.691	104.594
alpha. or .gamma. hexachlorocyclohexane	0.9999	0.120	0.400	103.477
Quintozene	0.9992	0.962	3.207	95.972
Heptachlor	0.9999	0.772	2.573	102.835
Aldri	0.9996	1.848	6.159	104.488
Heptachlor‐exo‐epoxide	0.9996	0.876	2.920	104.974
Heptachlor‐endo‐epoxide	0.9991	1.198	3.994	99.581
trans‐Chlordane	0.9995	4.056	13.519	102.960
o,p'‐DDE	0.9996	2.427	8.088	106.591
alpha‐Endosulfan	0.9995	1.600	5.333	105.718
cis‐Chlordane	0.9991	3.557	11.856	101.498
p,p'‐DDE	0.9997	2.345	7.818	105.143
Dieldrin	0.9994	3.217	10.724	103.759
o,p'‐DDD	0.9983	3.833	12.778	106.358
Endrin	0.9993	2.247	7.490	101.404
β‐Endosulfan	0.9989	3.253	10.844	99.353
p,p'‐DDD	0.9948	3.508	11.695	98.177
o,p'‐DDT	0.9997	3.992	13.307	98.538
p,p'‐DDT	0.9988	2.501	8.337	103.697
Methoxychlor	0.9990	2.864	9.546	103.010

Abbreviations: LODs, limit of detection; LOQs, limit of quantification; *r* ^2^, coefficient of determination.

**FIGURE 1:**
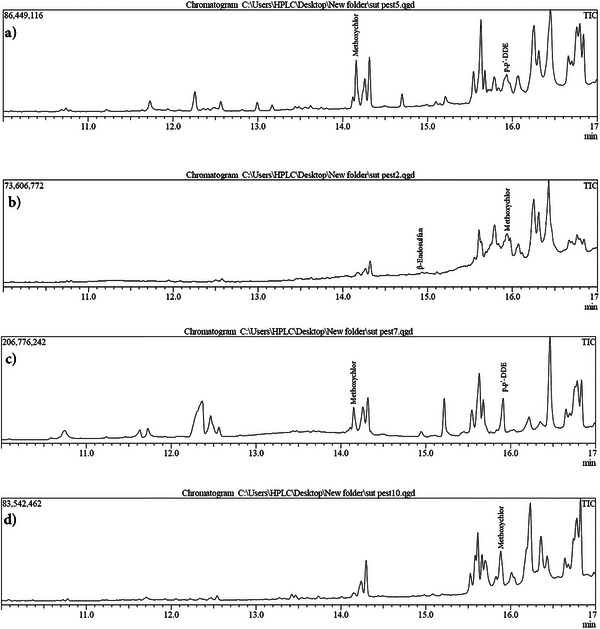
Representative chromatograms of milk samples in which methoxychlor, p,p'‐DDE and β‐Endosulfan were detected. (a) p,p'‐DDE; Retention time: 14.158, Concentration: 13.252 ppb. Methoxychlor; Retention time: 15.933, Concentration: 20.210 ppb, (b) β‐Endosulfan; Retention time: 14.847, Concentration: 3.847 ppb. Methoxychlor; Retention time: 15.936, Concentration: 21.338 ppb, (c) p,p'‐DDE; Retention time: 14.149, Concentration: 13.256 ppb. Methoxychlor; Retention time: 15.884, Concentration: 14.244 ppb, and (d) Methoxychlor; Retention time: 15.883, Concentration: 52.665 ppb.

**FIGURE 2:**
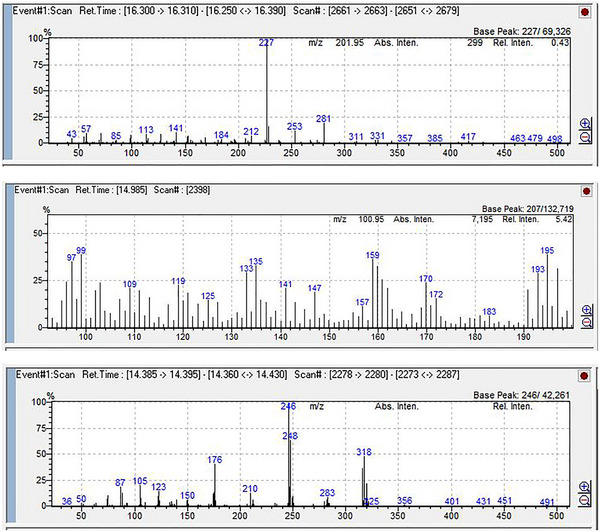
Ion chromatogram (a) methoxychlor, (b) β‐endosulfan, and (c) p,p'‐DDE.

Among the 23 pesticide residues screened in the residue analysis, only β‐endosulfan, methoxychlor, and p,p'‐DDE residues were determined (Tables 3 and 4; Figures 1 and 2). GC–MS system chromatograms of some milk samples in which residues were detected are presented in Figures 1 and 2.

### 3.2 Concentration of OCPs in Goat Raw Milk

A total of 23 OCP compounds were investigated in this study. A summary of the minimum, maximum, and average OCP concentrations and the number of positive samples is presented in Table 2. All samples were analyzed in triplicate, and the mean values were reported. OCP residues were detected in 14 of 20 goat milk samples. Among the 23 compounds screened, only three OCPs, β‐endosulfan, methoxychlor, and p,p'‐DDE, were detected. β‐Endosulfan and p,p'‐DDE were found in four samples, while methoxychlor was present in 14 samples.

**TABLE 2:** OCPs residue in goat's milk.

OCPs	Mean ± SE ppb	Range (min–max) ppb	MRL (mg/kg)	MRL (ppb)	Contaminated (*n*)	>MRL contaminated (*n*)
β‐Endosulfan	6.944 ± 2.036	2.233–11.858	0.05^*^	50	—	—
Methoxychlor	29.858 ± 4.332	14.244–69.403	0.01	10	14	11
p,p'‐DDE	9.947 ± 1.937	5.83–13.256	0.04^**^	40	—	—

Abbreviation: MRL; maximum residue limit.

* Endosulfan (α‐ and β‐isomers and total endosulfan sulphate in endosulfan) (F).

** DDT (total of p,p'‐DDT, o,p'‐DDT, p,p'‐DDE and p,p'‐TDE (DDD); in DDT) (F)″.

**FIGURE 3:**
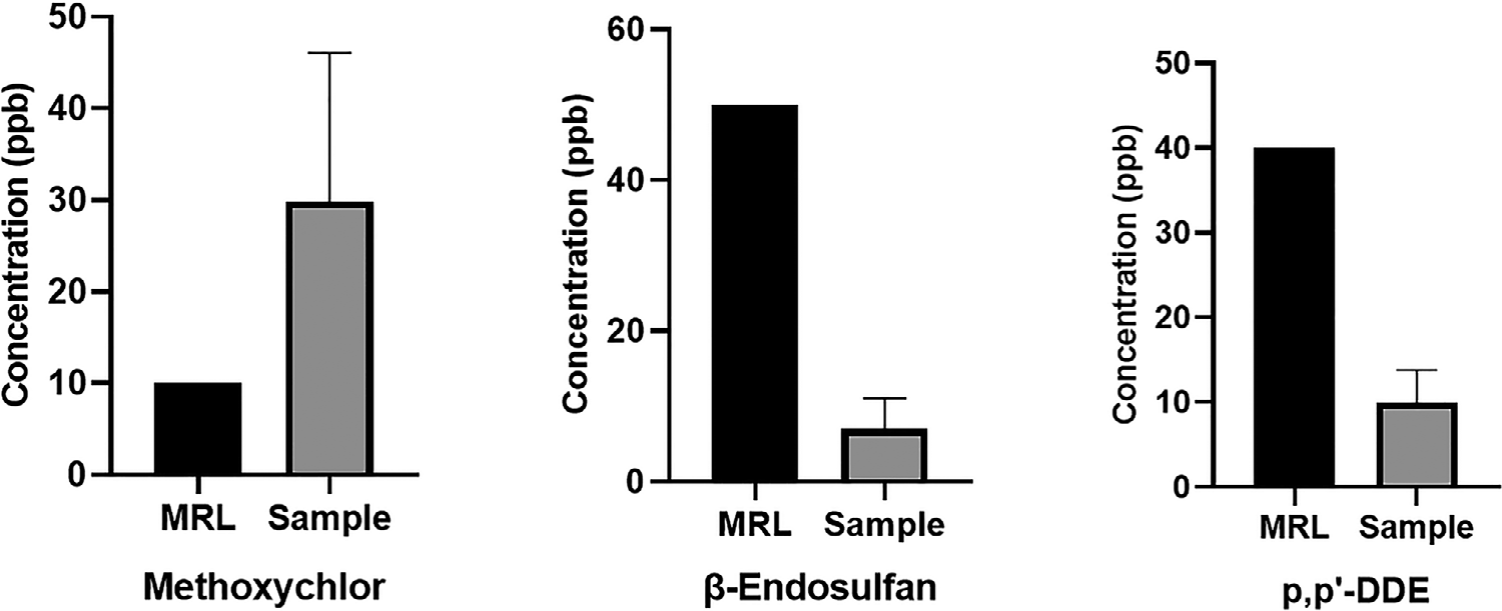
Data on pesticide residues in goat milk.

Table 2 and Figure 3 present the concentration ranges and maximum residue limits (MRLs) for the three organochlorine pesticide (OCP) residues detected in milk samples collected from various dairy farms in Kastamonu. Methoxychlor was the most frequently detected contaminant, found in 14 samples (70%), followed by β‐endosulfan and p,p'‐DDE, each detected in four samples (20%). The mean concentrations of methoxychlor, β‐endosulfan, and p,p'‐DDE in positive milk samples were 27.647 ± 4.899, 2.493 ± 0.495, and 9.306 ± 2.433 ppb, respectively.

The highest maximum concentration was observed for methoxychlor (69.403 ppb), followed by p,p'‐DDE (13.256 ppb), and β‐endosulfan (3.897 ppb). Among the 20 milk samples analyzed, six contained no detectable residues, 14 samples contained at least one or two different OCP residues, six samples had only one OCP detected, and eight samples had two different OCPs.

Methoxychlor contamination was detected in 14 of the analyzed milk samples, with 11 samples exceeding the MRL value of 0.01 ppb (Figure 3). Furthermore, β‐endosulfan (*n* = 4) and p,p'‐DDE (*n* = 4) contamination were detected, but none of the milk samples exceeded the MRL values. Notably, p,p'‐DDE is a major toxic metabolite of DDT, and its presence indicates potential historical or environmental contamination. p,p'‐DDE was detected in only four samples.

According to the Turkish Food Codex Regulation on Maximum Residue Limits of Pesticides (Turkish Food Codex, 2021), the maximum residue limit (MRL) in milk are set at 0.05 mg/kg (50 ppb) for β‐endosulfan, 0.01 mg/kg (10 ppb) for methoxychlor and 0.04 mg/kg (40 ppb) for p,p'‐DDE (1 ppb = 0.001 mg/kg, 1 mg/kg = 1000 ppb). In our study, the residue levels of β‐endosulfan and p,p'‐DDE were below the established MRL, while methoxychlor exceeded the MRL in 13 out of 14 positive samples (Table 4).

**FIGURE 4:**
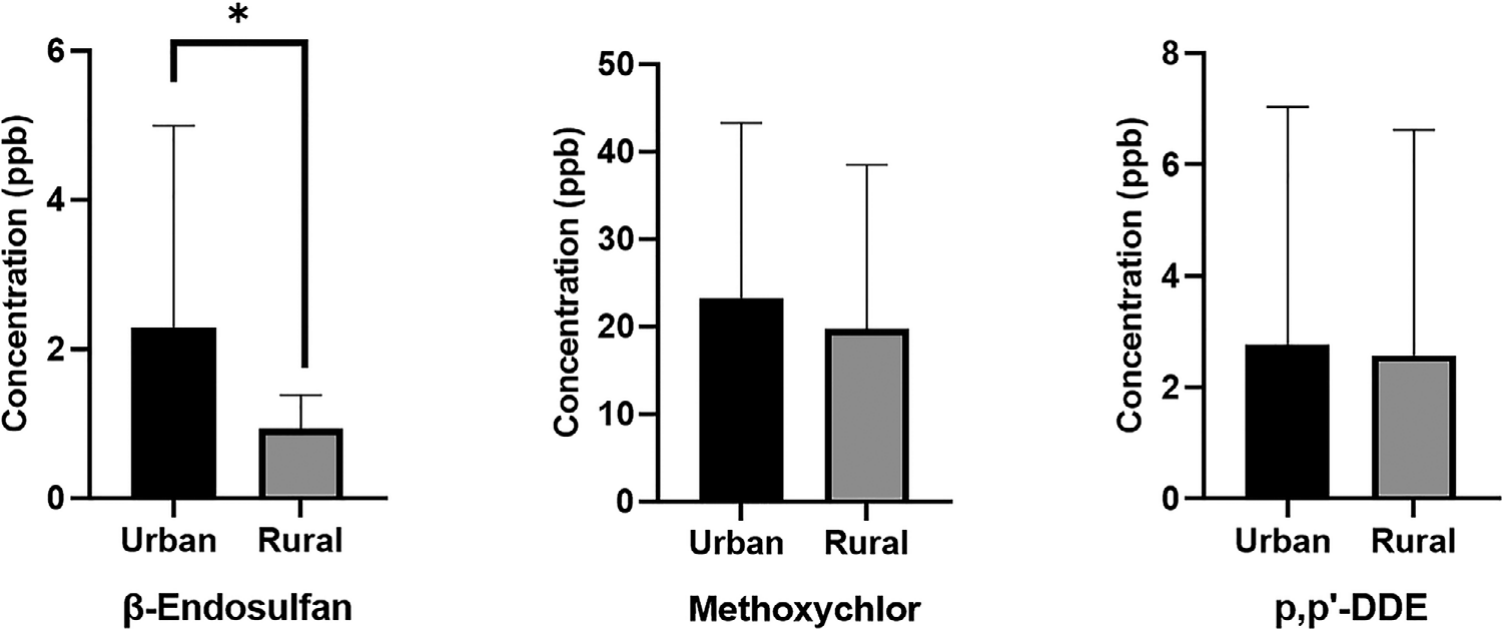
Pesticide residues detected in milk collected from goat farms in urban and rural areas (*p < 0.05).

In addition, the collected goat milk samples were categorized into two groups based on the geographical proximity of the farms to urban and rural settlements (Table 3; Figure 4). Farms located within 0–5 km of residential areas were classified as urban (*n* = 11), whereas farms located > 20 km away were classified as rural (*n* = 9). Although there were no statistically significant differences (*p* > 0.05) in methoxychlor and p,p'‐DDE residue levels between urban and rural farms, a statistically significant difference was observed for β‐endosulfan concentrations (*p* = 0.015). These findings suggest that goats are exposed to pesticide residues in both urban and remote areas of Kastamonu.

**TABLE 3:** Distribution of OCP residues in goat milk samples collected from urban and rural areas.

	Urban area (*n* = 11)		Rural area (*n* = 9)			Number of positive samples		Number of samples exceeding the MRL		Statistical analysis
OCP	Mean ± SE (ppb)	Min‐Max (ppb)	Mean ± SE (ppb)	Min–max (ppb)	MRL (ppb)	Urban area	Rural area	Urban area	Rural area	*p‐*value
β‐Endosulfan	5.305 ± 1.710	2.233–8.143	2.233	2.233	50	3	1	—	—	0.015
Methoxychlor	29.526 ± 6.873	16.150–69.403	30.190 ± 5.837	14.244–52.665	10	7	7	7	7	0.581
p.p'‐DDE	9.541 ± 3.711	5.830–13.252	10.352 ± 2.904	7.448–13.256	40	2	2	—	—	0.822

*Note*: For the calculation of *p*‐values, it is assumed that the undetectable data are half of the detection method limit (Aydin et al. 2019).

### 3.3 Risk Assessment

To estimate potential health risks to consumers, parameters such as LADD and HQ were calculated. Table 4 presents the implications of OCP residues on human health, focusing on milk from different farm goats. The results revealed that goat milk from all farms is considered safe for consumption and varies among farms of OCPs residues, especially β‐endosulfan, methoxychlor, and p,p’‐DDE. The results confirmed that the analyzed goat milk did not pose any health hazards to consumers, which might be caused by organochlorine xenobiotics. Interestingly, although methoxychlor was above the MRL, the hazard index (HI) remained below 1.

**TABLE 4:** Human health risk assessment of organochlorine pesticide (OCP) residues in raw goat milk.

	ADI (mg/kg)	LADD or EDI	RfD	HQ	Remarks
β‐Endosulfan	0.006 (∑Endosulfan)	1.84512 × 10^−^^5^	0.006	0.0030752	Goat milk is safe to consume
Methoxychlor	0.1	7.9337 × 10^−5^	0.005	0.015867394	Goat milk is safe to consume
p,p’‐DDE	0.01 (∑DDT)	2.64306 × 10^−5^	0.5	5.28612 × 10^−5^	Goat milk is safe to consume
Total HI	0.018995455 (<1)				

*Note*: HI = ∑HQ = 0.018995455, <1. ADI, admissible daily intake. EDI, estimated daily intake. ∑Endosulfan = α‐endosulfan+β‐endosulfan. ∑DDT = DDT, DDE, DDD. p,p'‐DD. The lifetime average daily dose (LADD), RfD (reference dose). HQ, hazard quotient.

## 4 Discussion

Türkiye is an agricultural country, and farmers use pesticides to increase their production. Various studies have been conducted to measure the use and effects of pesticides on health. The main objective of this study was to determine the levels of OCP contaminants in goat milk in Kastamonu/Türkiye, and to establish a baseline for chemicals for which such information was not previously available. This will enable an evaluation of future trends.

OCPs are a group of POPs that have been widely detected for years because of their strong insecticidal and weed‐killing properties, and have been overused, unconsciously and carelessly (Forbes et al. 2021). Today, their use is banned or significantly restricted worldwide. However, these compounds persist in the environment for decades, and exposure to them can cause neurological and endocrinological damage (Mrema et al. 2013; Sari and Esen 2022). In this study, 70% (*n* = 14) of the milk samples collected from milking tanks in 20 different goat farms contained at least one OCP compound, emphasizing their persistent presence in the environment. The detected OCPs were β‐endosulfan (*n* = 4), methoxychlor (*n* = 14), and p,p'‐DDE (*n* = 4). Feed sources may also play an important role in the transfer of pesticide residues into milk. In small ruminant production systems, goats are commonly fed with pasture vegetation, forage crops such as alfalfa and maize silage, cereal straw, and sometimes commercial compound feeds. Pesticide residues may accumulate in these feed materials depending on agricultural practices and environmental conditions. In addition, grazing animals may ingest small amounts of soil particles along with pasture vegetation, which may contain persistent pesticide residues. Therefore, feed origin and feeding practices should be considered as potential factors influencing pesticide contamination in goat milk (Sharma et al. 2014). Research and routine analyses to detect pesticide residues and their metabolites in a wide range of products, including animal feeds, fruits, and vegetables, have been conducted worldwide (Kumar et al. 2019; Mebdoua 2018). Pesticide residue studies have also been conducted in various body fluids and tissues, particularly in breast milk. The detection of pesticide residues in biological samples, such as milk, urine, and saliva, even in individuals not directly involved in agricultural work, indicates that exposure occurs through multiple pathways, most notably via dietary intake. The primary route of pesticide exposure in humans is the consumption of contaminated food. Among animal‐derived foods, milk, cheese, and butter, owing to their high fat content, are of particular concern for OCP exposure, as organochlorine pesticides tend to accumulate in fatty tissues (Mitro et al. 2015).

Today, no study has directly investigated pesticide residues in pasture environments, forage crops, or animal feed used for livestock in Kastamonu Province. However, several studies have examined pesticide residues in animal‐derived products. Aksoy et al. (2013) evaluated the levels of organochlorine pesticide (OCP) residues—including aldrin, hexachlorobenzene, 2,4‐DDE, 4,4‐DDE, 2,4‐DDT, 4,4‐DDT, and α‐, β‐, and γ‐HCH—in butter samples collected from the Black Sea region of Türkiye, including Kastamonu. The authors reported that the detected concentrations exceeded the legal limits established by the Turkish Food Codex. Considering the presence of OCP residues in butter samples, they emphasized the need for more extensive monitoring programs for organochlorine pesticides and other persistent pollutants in milk and dairy products in the Black Sea region for both environmental surveillance and public health protection.

Although DDT, endosulfan, and methoxychlor are included in the list of banned pesticides, all three metabolites were detected in raw goat milk. This contamination may be attributed to two primary factors: (1) illegal or unauthorized use in agricultural practices and (2) residual environmental contamination resulting from historical overuse (Langley and Alwasiyah 2025). These findings indicate that organochlorine pesticides (OCPs), despite regulatory restrictions, persist in the environment and can bioaccumulate in animal‐derived food products such as goat milk. The continued presence of these residues raises concerns about potential risks to public health. Therefore, proactive measures should be taken to safeguard consumer health, including increasing awareness among veterinarians and producers of pesticide contamination and its long‐term implications.

The results obtained are particularly noteworthy, especially for high levels of methoxychlor residue. The detected mean value of 29.858 ppb is approximately three times higher than the maximum residue limit of 10 ppb recommended by the FAO/WHO and Codex Alimentarius for dairy products. It was also found to be approximately seven times higher (69.403 ppb) in samples collected from some farms. Methoxychlor is an organochlorine insecticide similar to DDT and can remain in the environment for a long time. Because of its strong lipophilic properties, methoxychlor readily accumulates in the fatty tissues of animals and can be transferred to milk following ingestion of contaminated feed, water, or pasture (Tsiplakou et al. 2010; Witczak et al. 2016). Although the agricultural use of this pesticide was banned in Türkiye in 2009, its long persistence in the environment and its biological accumulation in lipid tissues continue to result in detectable residues in dairy products. Similarly, the p,p'‐DDE residue was notable at 9.947 ppb. p,p′‐DDE is the main degradation product of DDT and is formed through environmental and biological transformation processes. Even though DDT has been banned in Türkiye since 1985, its metabolites remain highly stable and can persist in soils, sediments, and food chains for decades (Dos Santos et al. 2015). The highest maximum concentration was observed for methoxychlor (69.403 ppb), followed by p,p′‐DDE (13.256 ppb) and β‐endosulfan (3.897 ppb). The predominance of methoxychlor may be explained by its higher lipid affinity and potential historical use in agricultural practices, which facilitate its accumulation in milk fat compared with other OCPs (Witczak et al. 2016). In contrast, the comparatively lower levels of β‐endosulfan may reflect differences in environmental degradation pathways and its relatively lower persistence in animal tissues compared with other organochlorine compounds (Singh et al. 2021).

Various national and international studies have reported the presence of organochlorine pesticide residues in goat milk. Kocabaş et al. (2016) reported that DDT derivatives and endosulfan residues were detected in goat milk samples, some of which exceeded the MRL limits. Similarly, Baydan et al. (2020) reported that p,p'‐DDE and DDD residues ranged from 3 to 7 ppb in milk samples collected from the Aegean Region. The p,p'‐DDE levels in our study were above these values and may indicate the presence of regional pesticide use or environmental contamination sources.

At the international level, Hernández et al. (2013) reported p,p'‐DDE levels ranging from 2.1 to 7.8 ppb in goat milk samples from Spain, while methoxychlor was not detected in most of the samples. Similarly, Singh et al. (2012) reported endosulfan residues at concentrations of 1–4 ppb in a study conducted in India. The fact that the values in our study exceeded these ranges suggests the persistence of local environmental contamination or the effects of past pesticide use.

Because organochlorine pesticides are highly stable in the environment, they can remain in the soil, water, and vegetation for many years. Pasture animals such as goats may ingest these residues directly from pastures or water sources. Owing to their lipophilic properties, these compounds accumulate in the adipose tissues of animals and can be transferred into milk. This is particularly concerning for children who are fed goat milk and dairy products, as organochlorine compounds can disrupt hormonal balance, affect liver enzyme function, and, in some cases, exhibit carcinogenic effects.

DDT transforms, resulting in metabolites, primarily dichlorodiphenyldichloroethane (DDD) and dichlorodiphenyldichloroethylene (DDE). While the half‐life of DDT in soil ranges from 2 to 15 years, it can reach up to 150 years in the aquatic environment. The predominant occurrence of the p,p'‐DDT isomer in milk samples is due to its persistence and lipophilicity and is an indication of its illegal use (Aydin et al. 2019). Commercial DDT consists of a mixture of several isomers, with the p,p’‐DDT isomer accounting for approximately 77% of the mixture. p,p’‐DDD and p,p'‐DDE are the most important metabolites resulting from the environmental degradation of DDT (Dos Santos et al. 2015). Although highly persistent organochlorine pesticides (OCPs) have been detected worldwide and in Turkey, highly persistent OCPs have been detected in cow and sheep milk (Aydin et al. 2019; Bulut et al. 2011; Güvenç and Aksoy 2010), there have been very few investigations of the residues in goat milk.

The detection of residues of several OCPs in goat milk demonstrates that animals are still exposed to these compounds, most likely through feeding. This is because many of these pesticides remain on the market or in storage and continue to be used even after they have been banned or persist in the environment after use (Vogt et al. 2012). In this study, the residue concentrations of DDE and β‐endosulfan were below the established MRLs, whereas methoxychlor levels exceeded the acceptable threshold. The results of our study indicate that the presence of banned OCPs in goat milk reflects the continued use of these pesticides by farmers and/or the persistence of residues in the environment. In the future, a continuous monitoring program is required to track the profiles of these compounds in animal milk and other environmental components.

When contamination levels in goat milk from rural and urban areas were evaluated by comparing the two groups, a significant difference was found only for β‐endosulfan (*p* = 0.015). However, there were no significant differences in the levels of methoxychlor and p,p'‐DDE in goat milk from rural and urban areas. This suggests that pesticide‐based agriculture is more intensive in urban areas. This difference may be related to variations in local agricultural activities and pesticide application patterns between the two environments. Farms located closer to urban settlements are often surrounded by more intensive crop cultivation, horticultural production, or small‐scale agricultural activities where pesticide application may occur more frequently. Consequently, pesticide residues such as endosulfan may reach grazing areas through spray drift, contaminated vegetation, soil particles, or surface runoff from nearby agricultural fields. In addition, organochlorine pesticides are known for their persistence in soil and water, which may lead to localized environmental contamination and differences in residue levels between farms in different geographical settings (K). Nevertheless, when considering the number of positive samples, the positivity rates for milk samples from both urban and rural areas were very similar.

Concerns about the potential effects of pesticide residues in milk on human health have increased due to the risks associated with cumulative exposure to multiple pesticides. In our study, although highly persistent OCPs were detected in contaminated milk, the amounts detected were not considered a health risk. The levels of DDT and β‐endosulfan in goat milk were below the MRLs, whereas methoxychlor levels exceeded the MRLs, raising concerns regarding human health.

## 5 Conclusion

In the present study, the concentration profiles and health risks associated with some common organochlorine pesticides (OCPs) in milk obtained from goat farms in Kastamonu were evaluated. The findings revealed that organochlorine pesticide residues persist in commonly consumed animal products, such as goat milk in Türkiye. Among the quantified OCPs, only methoxychlor exceeded the maximum residue limit (MRL) and was the predominant pesticide; compounds that have long been banned, their continued presence in the food chain is likely due to environmental persistence. Methoxychlor is a lipophilic organochlorine insecticide that can accumulate in fatty tissues and has been associated with endocrine‐disrupting effects and potential reproductive toxicity in humans following long‐term exposure. Therefore, soil and water analyses, feed audits, and regular milk testing are essential, particularly for pastoral areas where livestock graze directly on natural or semi‐natural pastures and may be exposed to environmental contaminants. It is also recommended that national pesticide monitoring programs be strengthened, that producers and consumers be made aware of this issue, and that pesticide analyses be conducted more frequently to ensure the safety of goat milk products. Future studies should aim to increase the statistical reliability by including more samples from different regions and assessing the seasonal variability of pesticide residues.

## Author Contributions

**Sedat Gökmen**: conceptualization, methodology, data curation, investigation, formal analysis, supervision, visualization, writing – original draft, writing – review & editing. **Nurcan Demir**: methodology, data curation.

## Funding

Part of this work was supported by The Scientific and Technological Research Council of Turkey (TÜBİTAK), under the 2209‐A Research Project Support Program for Undergraduate Students, Project No. 1919B012322491.

## Ethics Statement

An ethical statement is not required for this study.

## Conflicts of Interest

The authors declare no conflicts of interest.

## Data Availability

The data that support the findings of this study are available from the corresponding author upon reasonable request.
